# Circulating long-non coding RNAs as biomarkers of left ventricular diastolic function and remodelling in patients with well-controlled type 2 diabetes

**DOI:** 10.1038/srep37354

**Published:** 2016-11-22

**Authors:** D. de Gonzalo-Calvo, F. Kenneweg, C. Bang, R. Toro, R. W. van der Meer, L. J. Rijzewijk, J. W. Smit, H. J. Lamb, V. Llorente-Cortes, T. Thum

**Affiliations:** 1 Cardiovascular Research Center (CSIC-ICCC), Biomedical Research Institute Sant Pau (IIB Sant Pau), Barcelona, Spain; 2 Institute of Molecular and Translational Therapeutic Strategies (IMTTS), IFB-Tx, Hannover Medical School, Hannover, Germany; 3 Department of Medicine, University of Cádiz, Cádiz, Spain; 4 Department of Radiology, Leiden University Medical Center, Leiden, The Netherlands; 5 Department of Medicine, Kantonsspital Baden AG, Baden, Switzerland; 6 Department of Internal Medicine, University Medical Center Nijmegen, Nijmegen, The Netherlands; 7 Excellence Cluster REBIRTH, Hannover Medical School, Hannover, Germany; 8 National Heart and Lung Institute, Imperial College, London, UK

## Abstract

Contractile dysfunction is underdiagnosed in early stages of diabetic cardiomyopathy. We evaluated the potential of circulating long non-coding RNAs (lncRNAs) as biomarkers of subclinical cardiac abnormalities in type 2 diabetes. Forty-eight men with well-controlled type 2 diabetes and 12 healthy age-matched volunteers were enrolled in the study. Left ventricular (LV) parameters were measured by magnetic resonance imaging. A panel of lncRNAs was quantified in serum by RT-qPCR. No differences in expression levels of lncRNAs were observed between type 2 diabetes patients and healthy volunteers. In patients with type 2 diabetes, long intergenic non-coding RNA predicting cardiac remodeling (LIPCAR) was inversely associated with diastolic function, measured as E/A peak flow (P < 0.050 for all linear models). LIPCAR was positively associated with grade I diastolic dysfunction (P < 0.050 for all logistic models). Myocardial infarction-associated transcript (MIAT) and smooth muscle and endothelial cell-enriched migration/differentiation-associated long noncoding RNA (SENCR) were directly associated with LV mass to LV end-diastolic volume ratio, a marker of cardiac remodelling (P < 0.050 for all linear models). These findings were validated in a sample of 30 patients with well-controlled type 2 diabetes. LncRNAs are independent predictors of diastolic function and remodelling in patients with type 2 diabetes.

Cardiovascular complications are the leading cause of morbidity and death in patients with diabetes^1^. Diabetes affects cardiac structure and function in the absence of other comorbidities such as coronary disease or hypertension, a condition known as diabetic cardiomyopathy^2^. In recently diagnosed patients with well-controlled and uncomplicated type 2 diabetes, contractile dysfunction and adverse cardiac remodelling are subclinical events preceding the development of symptomatic heart failure (HF)^3^,^4^. We have demonstrated that intervention strategies could improve cardiac function in stable diabetic patients^5^. However, the detection of diabetic cardiomyopathy remains a challenge due to the absence of clinical symptoms in preclinical disease stages and the limited availability and relatively high costs of cardiac imaging techniques. Regular screening approaches could benefit from accurate, accessible and easy-to-apply diagnostic tools that expand current technologies used to detect early signs of cardiac alterations. As such, the development of diagnostic blood tests to predict and/or monitor cardiac abnormalities in type 2 diabetes patients is a key area of interest in the field of diabetes research.

Long non-coding RNAs (lncRNAs) are transcripts longer that 200 nucleotides that function as epigenetic regulators of gene expression^6^. Several lines of evidence indicate that lncRNAs play an essential role in cardiac development, function and disease^7^,^8^. Recent studies have proposed that lncRNAs can be used as cardiac biomarkers^9^,^10^. LncRNAs are highly cell- and context-specific, their expression correlates with cardiac status in both normal and pathological conditions and they can be detected in the extracellular fluids of patients^11^,^12^. Indeed, pioneering investigations recently identified circulating lncRNAs as useful diagnostic and prognostic biomarkers of cardiac remodelling and cardiovascular death^13^,^14^. Previous studies have examined the role of lncRNAs in diabetes-related cardiovascular complications *in vitro, in vivo* and in biopsied human tissue^15^,^16^. Little is known about the potential of circulating lncRNAs as indicators of diabetes and associated cardiovascular complications^17^. Here, we investigated for the first time the potential value of circulating lncRNAs as biomarkers of early cardiac alterations in patients with well-controlled type 2 diabetes. To this end, we evaluated a panel of lncRNAs directly involved in cardiovascular disease and/or diabetic conditions and their relationship with magnetic resonance imaging (MRI) indices of cardiac dimensions and function, in serum from patients with well-controlled type 2 diabetes of short duration.

## Results

### Circulating lncRNAs in type 2 diabetes patients and healthy controls

The clinical characteristics of the 60 participants included in the initial study are summarized in Table 1. A total of 48 type 2 diabetes patients and 12 age-matched healthy volunteers were enrolled. Body mass index (BMI) and systolic blood pressure (SBP) were higher in type 2 diabetes patients with respect to controls.

LncRNA analysis was performed on RNA derived from the serum of all type 2 diabetes patients and healthy volunteers. Long intergenic non-coding RNA predicting cardiac remodeling (LIPCAR), uc004cos.4, uc004cov.4, uc004coz.1, uc011mfi.2, uc022bqu.1, uc022bqw.1, HOX transcript antisense RNA (HOTAIR) and myocardial infarction-associated transcript (MIAT) were consistently amplified in all individual samples. H19 was below the limit of detection in 10% and 17% of type 2 diabetes and control samples, respectively. Smooth muscle and endothelial cell-enriched migration/differentiation-associated long noncoding RNA (SENCR) was below the limit of detection in 2% and 8% of type 2 diabetes and control samples, respectively. Both lncRNAs were used in further analyses. Metastasis-associated lung adenocarcinoma transcript 1 (MALAT1) levels were below the limit of detection in 65% and 75% of type 2 diabetes and control samples, respectively and this lncRNA was excluded from further analyses. Circulating levels of LIPCAR, uc004cos.4, uc004cov.4, uc004coz.1, uc011mfi.2, uc022bqu.1 and uc022bqw.1 were positively correlated with one other in both study groups (Supplementary Tables S1 and S2). A close correlation between circulating H19, HOTAIR, MIAT and SENCR levels was also observed in the type 2 diabetes group.

We next compared expression levels of lncRNAs in circulating from type 2 diabetes patients and age-matched healthy controls (Supplementary Figure S1). Levels of uc011mfi.2, uc022bqu.1, uc022bqw.1 were lower in type 2 diabetes patients as compared with healthy volunteers (*P* < 0.050 for all comparisons). In contrast, levels of MIAT were higher in type 2 diabetes patients as compared with healthy volunteers (*P* < 0.050). Nonetheless, no statistical differences in uc011mfi.2, uc022bqu.1, or uc022bqw.1 were observed between study groups after accounting for differences in BMI (*P* > 0.050 for all comparisons). No statistical differences in MIAT levels were detected between groups after adjusting for differences in BMI and SBP (*P* > 0.050).

### Association between circulating lncRNAs and left ventricular dimension and function parameters in patients with type 2 diabetes

Univariate linear regression analysis was performed to explore the associations between cardiac MRI markers of left ventricular (LV) dimension and function and circulating lncRNA levels (Table 2). Type 2 diabetes patients showed a significant association between indices of LV diastolic function and lncRNA expression levels. Positive associations were observed between: i) E-dec_peak_ and LIPCAR, uc004cos.4, uc004cov.4, uc004coz.1, uc022bqw.1 and SENCR; and ii) E-dec_mean_ and LIPCAR, uc004cos.4, uc004cov.4, uc004coz.1, uc011mfi.2, uc022bqu.1, uc022bqw.1 and SENCR. Inverse associations were observed between: i) E peak filling rate and LIPCAR; and ii) E/A peak flow ratio and LIPCAR, uc004cos.4, uc004cov.4, uc004coz.1, c011mfi.2, uc022bqu.1 and uc022bqw.1. As regards cardiac structure, LVMV-ratio was positively associated with MIAT and SENCR expression. No significant association was observed between either H19 or HOTAIR and any of the parameters of LV diastolic function and structure analysed (*P* > 0.050 for all associations). There were no associations identified between circulating lncRNA levels and LV dimension, systolic function, or myocardial steatosis, except for MIAT, which showed a weak inverse association with LV end-diastolic volume in type 2 diabetes patients. In healthy volunteers, no association between cardiac MRI parameters and circulating lncRNA levels was observed (*P* > 0.050 for all associations).

### Association between circulating lncRNAs and clinical characteristics in type 2 diabetes patients

To further explore the potential of circulating lncRNAs as predictors of cardiac dimensions and function, we evaluated the extent to which lncRNA levels were influenced by clinical characteristics in type 2 diabetes patients (Table 3). HDL-C and time since diagnosis of diabetes were positively associated with expression levels of the mitochondrial-derived lncRNAs LIPCAR, uc004cos.4, uc004cov.4, uc004coz.1, uc011mfi.2, uc022bqu.1 and uc022bqw.1. By contrast, expression levels of these same lncRNAs were inversely associated with BMI, waist circumference, plasma fasting insulin and subcutaneous fat volume. Age was positively associated with levels of LIPCAR, uc004cos.4, uc004cov.4 and uc022bqw.1. us-CRP was negatively associated with LIPCAR, uc004cov.4, uc011mfi.2, uc022bqu.1 and uc022bqw.1 expression. No association was detected between any other clinical characteristics and the expression of these lncRNAs. There was no significant association between H19, HOTAIR, MIAT and SENCR and any of the clinical characteristics studied (*P* > 0.050 for all associations). Circulating levels of the lncRNAs analysed were not significantly associated with the use of statins, antihypertensive medication, β-blockers, diuretics, angiotensin-converting enzyme (ACE) inhibitors, angiotensin receptor blockers (ARBs), or calcium antagonists (*P* > 0.050 for all associations).

### Circulating LIPCAR is an independent predictor of left ventricular diastolic function in patients with type 2 diabetes

E/A peak flow was used as measure of LV diastolic function for further analyses^18^,^19^,^20^. Given that expression levels of LIPCAR, uc004cos.4, uc004cov.4, uc004coz.1, uc011mfi.2, uc022bqu.1 and uc022bqw.1 were positively correlated with each other, we selected LIPCAR, the lncRNA most strongly associated with E/A peak flow, for subsequent statistical analysis.

Multivariate analysis was performed to explore in detail the relationship between LV diastolic function and LIPCAR levels in patients with well-controlled type 2 diabetes of short duration (Table 4). To this end, E/A peak flow was entered as a dependent variable and age, BMI and LIPCAR expression were entered as independent variables (model 1). Neither age nor BMI had any effect on the association between E/A peak flow and LIPCAR. Time since diagnosis of diabetes, plasma fasting insulin, HDL-C and us-CRP, all of which were significantly associated with circulating LIPCAR levels in univariate analysis, and plasma fasting glucose, myocardial steatosis, SBP, diastolic blood pressure (DBP) and heart rate, which have been previously associated with diastolic function^19^, were separately entered as independent variables into model 1 (model 2), in order to take into account the potential confounding effect of these variables on the associations observed. The established clinical biomarker NT-proBNP was also entered separately as an independent variable into the same model for a more detailed study of circulating lncRNAs as potential biomarker. Adjustment for different clinical, biochemical, or metabolic variables had no effect on the association between diastolic function and circulating LIPCAR levels. To gain further insight into the association between LV diastolic function and LIPCAR we compared E/A peak flow levels in patients in the highest quartile of LIPCAR expression with those in all other quartiles (Supplementary Figure S2A). E/A peak flow levels were significantly lower in patients in the highest quartile compared with those in quartiles 1, 2 and 3 (*P* < 0.050).

Given the observed association between LV diastolic function parameters and LIPCAR, uc004cos.4, uc004cov.4, uc004coz.1, c011mfi.2, uc022bqu.1 and uc022bqw.1 expression levels, we hypothesized that circulating levels of these lncRNAs could constitute potential biomarkers of LV diastolic impairment in patients with stable type 2 diabetes. We stratified the type 2 diabetes population according to current recommendations for LV diastolic function^21^, thus forming 2 groups: type 2 diabetes patients with normal diastolic function (N = 24) and type 2 diabetes patients with grade I diastolic dysfunction (N = 24). The prevalence of diastolic dysfunction (50%) was comparable with that reported in previous studies^2^. Univariate logistic regression revealed that LIPCAR, uc004cos.4, uc004cov.4, uc004coz.1, c011mfi.2, uc022bqu.1 and uc022bqw.1 were predictors of grade I diastolic dysfunction (Table 5). LIPCAR was most strongly associated with LV diastolic function parameters and was thus used in further analyses. Multivariate logistic regression models identified LIPCAR as an independent predictor of grade I diastolic dysfunction in patients with type 2 diabetes, even after adjusting for age and BMI (model 1) and other potential confounding factors (model 2) (Table 5). The models showed good discrimination: area under the ROC curve (AUC) from 0.745 (0.559, 0.891) to 0.842 (0.725, 0.959) (Supplementary Table S3). Furthermore, type 2 diabetes patients with abnormal diastolic dysfunction showed higher levels of LIPCAR as compared with patients with normal diastolic function (*P* < 0.050) (Supplementary Figure S2B). Neither E/A peak flow nor grade I diastolic dysfunction were associated with levels of the established biomarkers NT-proBNP or us-CRP (*P* > 0.050 for all associations).

### Circulating MIAT and SENCR as independent predictors of left ventricular remodelling in patients with type 2 diabetes

Univariate linear regression analysis revealed a strong association between LVMV-ratio and circulating MIAT and SENCR levels. To further assess the potential of these lncRNAs as predictors of LV remodelling, the association between LVMV-ratio and circulating MIAT and SENCR levels was explored using multivariate linear regression models (Table 6). In model 1, LVMV-ratio was entered as a dependent variable and age, BMI and MIAT or SENCR levels as independent variables. The following possible confounders were separately entered as independent variables into model 1 (model 2): plasma fasting glucose, plasma fasting insulin, time since diagnosis of diabetes, heart rate, SBP, DBP, myocardial steatosis, us-CRP and NT pro-BNP. Circulating MIAT levels were independently associated with LVMV-ratio after adjusting for all confounders. Similar results were obtained for SENCR. Type 2 diabetes patients in the fourth quartile of circulating MIAT or SENCR levels showed a higher LVMV-ratio than those in lower quartiles (*P* < 0.050) (Supplementary Figure S2C). No association was observed between LVMV-ratio and levels of NT-proBNP or us-CRP (*P* > 0.050 for all associations).

### Validation study

Circulating levels of LIPCAR, SENCR and MIAT were further evaluated in a validation study of 30 men with well-controlled type 2 diabetes (Table 7). In agreement with the findings presented above, type 2 diabetes patients showed a significant association between the index of LV diastolic function E/A peak flow and circulating LIPCAR expression levels (Table 8). As expected, E/A peak flow levels were significantly lower in patients in the third tertile compared with those in tertiles 1 and 2 (Supplementary Figure S3A). A direct association between LVMV-ratio and circulating MIAT and SENCR levels was also observed in the validation study (Table 8). Furthermore, patients in the third tertile of circulating MIAT or SENCR levels showed a higher LVMV-ratio than those in lower tertiles (Supplementary Figure S3B). Finally, confirming the results of our initial study, no association was observed between E/A peak flow, grade I diastolic dysfunction or LVMV-ratio and levels of NT-proBNP or us-CRP in the validation study (*P* > 0.050 for all associations).

## Discussion

In a population-based study of patients with well-controlled type 2 diabetes, we demonstrated that circulating lncRNA levels are independent predictors of LV diastolic function and remodelling. To our knowledge this is the first study to investigate the potential of circulating lncRNAs as biomarkers of cardiovascular complications in diabetes.

Previous studies have produced evidence of subclinical LV diastolic dysfunction and adverse LV concentric remodelling in patients with uncomplicated type 2 diabetes^22^,^23^. Both conditions are preclinical events in the pathophysiology of diabetic cardiomyopathy that are intimately associated with adverse cardiovascular prognosis, including cardiovascular death^23^. Improvement in LV compliance could be achieved through the implementation of therapeutic interventions, particularly in subclinical stages of the disease^5^. However, the diagnosis of early cardiac dysfunction in type 2 diabetes remains challenging. The identification of novel blood biomarkers with which to monitor diastolic function and cardiac remodelling is of major clinical importance for the correct management of diabetic patients. Non-coding RNA-based approaches provide new opportunities to develop novel biomarkers. Small RNAs such as microRNAs (miRNAs) have been proposed as sensitive and specific biomarkers of cardiovascular disease^24^,^25^. Extracellular lncRNAs are potent biomarkers in cancer^26^. Indeed, assays with clinical application have been developed for the detection of urine PCA3, a lncRNA reported to be a highly specific biomarker of prostate cancer^27^. Nonetheless, no previous study has investigated the potential use of circulating lncRNAs as indicators of early cardiac abnormalities in type 2 diabetes. We evaluated a panel of lncRNAs implicated in cardiovascular disease and/or diabetic conditions in relation with a wide range of measures of LV dimension and function in asymptomatic patients with well-controlled type 2 diabetes. First, we found that expression levels of all mitochondrial-derived lncRNAs evaluated were inversely correlated with parameters of LV diastolic function, with the strongest association observed for LIPCAR. Circulating LIPCAR levels were inversely correlated with the marker of LV diastolic function E/A peak flow, independently of possible confounders. Using a different approach, we demonstrated that LIPCAR is an independent predictor of grade I diastolic dysfunction in patients with no clinically detectable cardiac alterations. These results confirm and expand upon previous findings identifying circulating LIPCAR as a biomarker of chronic HF or adverse outcome after MI^13^. Second, the direct association of LVMV-ratio with circulating MIAT and SENCR, even after adjusting for possible confounding factors, indicates that both lncRNAs are independent predictors of LV remodelling in patients with stable type 2 diabetes. Corroborating these associations, higher LVMV-ratios, indicating increased LV concentric remodelling, were found in patients in the highest quartiles of MIAT and SENCR expression. Validation studies in a second population of patients with type 2 diabetes confirmed these findings. Interestingly, the association between circulating lncRNAs and LV function and remodelling appears to be specific to type 2 diabetes patients, as no such correlation was observed for the control group. Our data demonstrate that circulating levels of LIPCAR and MIAT or SENCR may constitute novel biomarkers of LV diastolic function and remodelling, respectively, in patients with well-controlled type 2 diabetes.

Exploration of the associations between of LV dimension and function parameters and existing cardiovascular biomarkers, such as NT-proBNP and us-CRP, revealed additional value of circulating lncRNAs as biomarkers. Neither of the 2 biomarkers were associated with LV function or remodelling parameters. The potential clinical utility of lncRNAs was thus superior to that of available cardiac biomarkers. Furthermore, the association between LV diastolic function and remodelling and the lncRNAs analysed revealed remarkable stability after adjustment for other predictors. These findings indicate that lncRNAs provide additional information over standard clinical diagnostic tests in asymptomatic type 2 diabetes patients.

Mitochondria-derived lncRNAs were associated with age, body composition parameters, metabolic parameters and inflammation. Specifically, LIPCAR was strongly correlated with waist circumference, plasma fasting insulin, subcutaneous fat volume and HDL-C. These results suggest that LIPCAR levels are associated with metabolic homeostasis, which could limit its value as a specific biomarker of cardiac impairment. Nonetheless, as noted previously, these clinical characteristics had no influence on the independent relationship between LV diastolic function and LIPCAR expression. Notably, MIAT and SENCR levels were not correlated with other clinical, biochemical, or metabolic parameters, supporting the use of these lncRNAs as biomarkers of LV remodelling.

Importantly, no differences in circulating expression levels of lncRNAs were observed between diabetic and non-diabetic participants. Although unadjusted analyses revealed contrasting uc011mfi.2, uc022bqu.1, uc022bqw.1 and MIAT levels between groups, these differences did not persist after adjusting for body composition and blood pressure and were thus attributable to divergent clinical characteristics between groups.

We can only speculate as to the biological implications of the associations observed. The association between LV function and remodelling and circulating levels of lncRNAs in type 2 diabetes patients with verified absence of structural heart disease or cardiac ischemia, even after adjustment for possible confounding factors, suggests that circulating levels of these lncRNAs may be at least in part linked to diabetic cardiomyopathy or related conditions. Mitochondrial dysfunction is implicated in the aetiology of diabetic cardiomyopathy^28^ and transcriptional profiling has revealed an abundance of mitochondria-derived lncRNAs in human LV samples^10^. Both these observations support the close correlation described here between circulating LIPCAR levels and LV diastolic dysfunction in type 2 diabetes patients. In addition, the association observed between both MIAT and SENCR, which are implicated in vascular conditions^16^,^29^ and LVMV-ratio, a marker of cardiac remodelling, is consistent with the reported relationship between microvascular dysfunction and LV cardiac remodelling^30^. Indeed, microvascular alterations could not be excluded by dobutamine stress echocardiography in our study participants^19^.

Strengths of our study are the considerable number of patients/subjects that have been analysed using MRI and the control of potential confounding factors, including structural disease and ischemia. Some limitations of the present study should be noted. First, generalization of the results is somewhat limited by the defining characteristics of the type 2 diabetes population: men with well-controlled disease and no significant comorbidities. Second, the panel of lncRNAs examined is biased by subjective selection. Future studies using global transcriptomics analyses will identify novel lncRNAs with potential as biomarkers of diabetes-related cardiac complications. Third, despite the robustness of the associations observed here a larger sample size would have been desirable. The strict inclusion and exclusion criteria, as well as the techniques used (e.g. MRI) limited the sample size. Fourth, our findings need to be validated in larger independent populations. Fifth, we could not determine whether the observed associations imply causal relationships. Sixth, our data do not allow us to identify the tissue origin of circulating lncRNAs. Seventh, the biological role of these lncRNAs in cardiac physiology or pathology remains unknown. Finally, a synthetic exogenous miRNA derived from *Caenorhabditis elegans*, cel-miR-39, was used as internal control. Differences related to RNA size during RNA isolation should be taken into account. There is a lack of accepted internal standards for extracellular non-coding RNAs, including lncRNAs. The use of different methods (e.g. lncRNAs with stable expression, lncRNAs lacking sequence homology to human lncRNAs spiked in the samples or the Ct average, among others) should improve the normalization of the data^31^.

Despite these limitations, our study provides the first insight into the potential value of circulating lncRNAs as indicators of cardiovascular conditions in patients with type 2 diabetes. We demonstrate that circulating lncRNAs are independent predictors of LV diastolic function and remodelling in asymptomatic patients with well-controlled type 2 diabetes. Circulating lncRNAs thus emerge as promising biomarkers of preclinical LV impairment in diabetic cardiomyopathy. A simple blood test based on LIPCAR, MIAT and SENCR could serve as a useful early diagnostic tool for the assessment of LV diastolic function and remodelling in patients with type 2 diabetes. Additional studies are required to validate these findings and provide further data into the potential role of circulating lncRNAs as biomarkers of cardiac complications in type 2 diabetes.

## Methods

### Subjects

Clinical and demographic data on all patients participating in the present study have been previously published as part of the PIRAMID (Pioglitazone Influence on triglyceride Accumulation in the Myocardium In Diabetes) study^5^, a prospective multicentre study designed to evaluate the effect of pioglitazone on myocardial function in 78 patients with well-controlled type 2 diabetes of short duration and with verified absence of structural heart disease or inducible ischemia. Men with uncomplicated type 2 diabetes aged 45 to 65 years were eligible. Patients were recruited by advertisement in local newspapers. The inclusion criteria were as follows: (i) glycated haemoglobin (HbA_1c_) level between 6.5% and 8.5%; (ii) BMI between 25 and 32 kg/m^2^; (iii) SBP < 150 mmHg/DBP < 85 mmHg, with or without the use of antihypertensive drugs. The exclusion criteria were: (i) any clinically significant disorder, particularly any history or complaints of cardiovascular or liver disease, or diabetes-related complications, including proliferative retinopathy, autonomic neuropathy, as excluded by Ewing’s test^32^, microalbuminuria, as excluded by determination of urine albumin/creatinine ratio, substance abuse and all contraindications to MRI; and (ii) prior use of thiazolidinediones or insulin. High-dose dobutamine stress echocardiography was performed to exclude cardiac ischemia or arrhythmias. The 78 patients were randomly distributed between the initial (N = 48) and validation groups (N = 30). Randomization was blinded to patient characteristics. Differences in age, waist circumference, plasma fasting glucose and diuretic use were observed between initial and validation groups (Supplementary Table S4).

Serum samples from 12 male control volunteers within the same range of age (45–65 years) were obtained from a previous study^19^. As the patient group these subjects were recruited by advertisements in the local newspapers. Control subjects were required to fulfil the following inclusion criteria: no known acute or chronic disease based on medical history, physical examination and standard laboratory tests (blood counts, fasting blood glucose, lipids, serum creatinine, liver enzymes and electrocardiogram). Exclusion criteria were as follows: (i) chronic use of any drug; (ii) substance abuse; (iii) hypertension; (iv) impaired glucose tolerance (as excluded by a 75-g oral glucose tolerance test). The study was performed at two institutes in The Netherlands (Leiden University Medical Center, Leiden, and Vrije Universiteit Medical Center, Amsterdam) and approved by both local ethics committees. Written informed consent was obtained from all participants. This study was performed in full compliance with the Declaration of Helsinki.

Blood sampling, study procedures and laboratory analyses have been previously described^5^,^19^,^33^.

### Magnetic resonance imaging

MR assessments in both study groups were performed at a single site (Leiden) using a 1.5-T whole-body MR scanner (Gyroscan ACS/NT15; Philips, Best, the Netherlands). The entire heart was imaged in the short-axis orientation with ECG-gated breath-hold balanced steady state free-precession imaging^34^. The following measures of cardiac dimensions were determined: LV mass; LV end-systolic volume; LV end-diastolic volume; and stroke volume. LV mass/volume ratio (LVMV-ratio) was calculated as the LV mass to LV end-diastolic volume ratio. Measures of systolic function were LV ejection fraction and cardiac index (cardiac output/body surface area). An ECG-gated gradient echo sequence with velocity encoding was performed to measure blood flow across the mitral valve to determine LV diastolic function parameters, including peak filling rates of the early filling phase (E), atrial contraction (A) and the E/A ratio. Peak (E-dec_peak_) and mean (E-dec_mean_) deceleration gradients of the E wave were also calculated^34^. LV filling pressures (E/Ea) were estimated^35^. Diastolic function was classified according to current European Association of Echocardiography and American Society of Echocardiography guidelines^21^. Patients were divided into 2 categories: normal diastolic function and grade I diastolic dysfunction groups, after ruling out the presence of pseudonormal or restrictive patterns. Images were analysed quantitatively using dedicated software (MASS and FLOW, Medis, Leiden, the Netherlands).

### RNA isolation and quantification of lncRNAs

LncRNA quantitative analysis was restricted to a panel of 12 lncRNAs previously associated with cardiovascular disease and/or diabetic conditions. Specifically, we analysed LIPCAR, uc004cos.4, uc004cov.4, uc004coz.1, uc011mfi.2, uc022bqu.1 and uc022bqw.1, all of which have been associated with LV remodelling post myocardial infarction^13^. These lncRNAs are of mitochondrial origin and are therefore highly expressed in the heart^10^. MALAT1 and MIAT have been implicated in microvascular complications of DM^15^,^16^. Notably, MALAT1 regulates hyperglycaemia-induced inflammation in endothelial cells^36^ and MIAT participates in the signaling pathway for high glucose-induced renal tubular epithelial injury^37^. Circulating MALAT1 and MIAT are univariate predictors of LV dysfunction after MI^14^. Circulating MALAT1 was associated with DM in MI patients^14^. Although a causal relationship has not been established, genetic variation in MIAT has been proposed as a risk factor for MI^38^. H19 has been implicated in the control of metabolic alterations induced by type 2 diabetes^39^ and upregulation of cardiac H19 expression has been reported in a mouse model of HF^40^. HOTAIR is implicated in aortic valve calcification^41^. Finally, SENCR has been proposed as a vascular-enriched lncRNA involved in the control of smooth muscle cell phenotype^29^. SENCR is highly expressed in tissues enriched in human smooth muscle cells and endothelial cells, including the heart^29^.

Total RNA extraction was performed from serum samples (200 μl) using *miRNeasy Serum*/*Plasma Kit* (Qiagen), according to the manufacturer’s instructions. Synthetic *Caenorhabditis elegans* miR-39-3p (cel-miR-39-3p) was added as an external standard (1.6 × 10^8^ copies/μL). Cel-miR-39-3p was spiked into samples during RNA isolation after incubation with the denaturing solution. Quality of the isolated RNA was evaluated using Agilent 2100 Bioanalyzer (Agilent Technologies). We have previously demonstrated that extracellular lncRNA are stably detected with no effect of room temperature (up to 24 hours) or repetitive freeze/thaw cycles (four cycles)^13^. cDNA was synthesized using the iScrip select cDNA synthesis kit (Bio-Rad). LncRNAs were analysed by amplification via RT-qPCR (iQ™ SYBR® Green Supermix, Bio-Rad) using a CFX-384 Bio-Rad machine. Primer sequences of the lncRNAs analysed are shown in Supplementary Table S5. The NCBI browser (http://www.ncbi.nlm.nih.gov/tools/primer-blast/), Primer 3 (http://primer3.ut.ee/) and Oligo Calc (http://biotools.nubic.northwestern.edu/OligoCalc.html) were used to design primers for the specific lncRNAs. The primer sequences for LIPCAR, uc004cos.4, uc004cov.4, uc004coz.1, uc011mfi.2, uc022bqw.1 and uc022bqu.1 were previously described by our group^13^. Specific primer sequences for MIAT^42^ and SENCR^29^ were also described previously. The detection of specific lncRNAs was validated by conventional PCR and agarose gel electrophoresis. Optimal annealing temperature was tested and optimized in advance for each primer pair. The CFX Manager 3.1 software (Bio-Rad) was used for both the determination of the threshold cycle (Ct) and for the melting curve analysis. The Ct was defined as the fractional cycle number at which the fluorescence exceeded a given threshold. The specificity of the PCR reaction was evaluated by melting curve analysis. LncRNA were considered to be expressed when Ct values were less than 39 and were detected with 3 Ct less than the negative control. This approach has been previously used in the field of circulating non-coding RNAs as potential clinical biomarkers^43^. LncRNAs in which 80% of the samples did not meet these criteria were considered to be below the limit of detection. The normalization control cel-miR-39 was amplified by specific TaqMan assay (Applied Biosystems). We have previously used this approach for blood-based lncRNAs^13^,^44^. Relative quantification was performed using the 2^−dCt^ method, where dCt = mean Ct_target_ − mean Ct_cel-miR-39_.

### Statistical analysis

Data are presented as mean ± SD for continuous variables and as frequencies (percentages) for categorical variables. Data normality was evaluated using the Kolmogorov-Smirnov test. Non-normally distributed variables (circulating lncRNAs, heart rate, fasting plasma glucose, plasma triglycerides, us-CRP and NT-proBNP) were logarithmically transformed to account for nonlinearity. Continuous variables were compared between groups using a Student’s t-test for independent samples. Circulating lncRNA expression levels were also adjusted for BMI and SBP with ANCOVA models to account for differences between type 2 diabetes and control groups. Correlations between variables were analysed using Pearson’s correlation analysis and the results presented using Pearson’s correlation coefficient (ρ). Linear regression analyses were performed to detect associations between clinical characteristics or cardiac MRI parameters and lncRNA expression. To identify independent predictors of cardiac function and remodelling in multivariate analyses, E/A peak flow or LVMV-ratio were entered into the models used as a dependent variable and circulating lncRNAs, age and BMI were subsequently entered as independent variables (model 1). In addition, possible confounding variables such as plasma fasting glucose, plasma fasting insulin, time since diagnosis of diabetes, SBP, DBP, heart rate, myocardial steatosis, us-CRP and NT-proBNP were separately entered into the model 1 (model 2). Results are expressed as standardized beta (β) coefficients. Logistic regressions were analysed to explore the association between circulating lncRNAs and diastolic dysfunction as outcome. Diastolic dysfunction was entered as a dependent variable and subsequently, circulating lncRNAs, age and BMI were as independent variables (model 1). In order to establish whether the observed association between diastolic dysfunction and circulating lncRNAs expression levels could be influenced by possible confounders, model 1 was also adjusted for plasma fasting glucose, plasma fasting insulin, time since diagnosis of diabetes, SBP, DBP, heart rate, myocardial steatosis, us-CRP or NT-proBNP (model 2). The results are presented as the odds ratio (OR) and 95% confidence intervals (CI). The AUC was analysed to explore the accuracy of the logistic regression models. All potential confounding factors were selected based on previous observations from our group and others^19^,^45^, or results observed in current investigation. Values of *P* < 0.050 were considered statistically significant. All statistical analyses were performed using the statistical software package SPSS 15.0 for Windows (SPSS Inc).

## Additional Information

**How to cite this article**: de Gonzalo-Calvo, D. *et al*. Circulating long-non coding RNAs as biomarkers of left ventricular diastolic function and remodelling in patients with well-controlled type 2 diabetes. *Sci. Rep.* **6**, 37354; doi: 10.1038/srep37354 (2016).

**Publisher’s note:** Springer Nature remains neutral with regard to jurisdictional claims in published maps and institutional affiliations.

## Supplementary Material

Supplementary material

## Figures and Tables

**Table 1:** Characteristics of the study population.

Variable	Type 2 diabetes Patients	Healthy Subjects	*P*-value
	N = 48	N = 12	
Age (years)	57.5 ± 5.4	57.7 ± 6.7	0.940
Time since diagnosis of diabetes (years)	4.0 ± 2.5	—	
Body mass index (kg/m^2^)	29.2 ± 3.5	26.8 ± 2.1	0.005*
Waist circumference (cm)	106.4 ± 9.8	98.8 ± 8.9	0.021*
Subcutaneous fat volume (mL)	706.6 ± 248.4	558.6 ± 209.7	0.063
Visceral fat volume (mL)	444.65 ± 206.6	324.5 ± 125.2	0.060
Systolic blood pressure (mm Hg)	128.1 ± 12.2	112.6 ± 9.3	<0.001*
Diastolic blood pressure (mmHg)	75.9 ± 6.1	76.2 ± 9.0	0.931
Heart rate (bpm)	65.7 ± 8.5	61.1 ± 9.8	0.082
Concomitant medication N (%)			
Statin	25 (52.1)	—	
Any antihypertensive medication	21 (43.8)	—	
β-Blocker	6 (12.5)	—	
Diuretic	11 (22.9)	—	
ACE inhibitor	10 (20.8)	—	
ARB	5 (10.4)	—	
Calcium antagonist	3 (6.3)	—	
HbA_1c_ (%)	7.2 ± 1.0	—	
Plasma fasting glucose (mmol/L)	9.0 ± 2.1	5.3 ± 0.4	<0.001*
Plasma fasting insulin (pmol/L)	66.8 ± 35.4	42.6 ± 22.2	0.009*
Total cholesterol (mmol/L)	4.6 ± 0.9	5.1 ± 0.5	0.114
LDL cholesterol (mmol/L)	2.7 ± 0.8	3.3 ± 0.5	0.022*
HDL cholesterol (mmol/L)	1.1 ± 0.3	1.3 ± 0.2	0.057
Plasma triglycerides (mmol/L)	1.8 ± 1.1	1.1 ± 0.5	0.017*
Plasma NEFA (mmol/L)	0.5 ± 0.2	0.5 ± 0.2	0.990
us-CRP (mg/L)	5.3 ± 4.2	6.3 ± 3.7	0.261
NT-proBNP (ng/L)	38.4 ± 29.8	36.6 ± 24.2	0.952

Data are presented as mean ± SD for continuous variables and as frequencies (percentages) for categorical variables. *Statistically significant. ACE: Angiotensin-converting enzyme; ARB: angiotensin receptor blocker; HbA_1c_: Glycated haemoglobin. For other abbreviations see the text.

**Table 2:** Associations between parameters of cardiac dimensions and function and circulating lncRNAs in patients with well-controlled type 2 diabetes.

	mean ± SD	LIPCAR		uc004cos.4		uc004cov.4		uc004coz.1		uc011mfi.2		uc022bqu.1		uc022bqw.1		MIAT		SENCR	
		β	*P*-value	β	*P*-value	β	*P*-value	β	*P*-value	β	*P*-value	β	*P*-value	β	*P*-value	β	*P*-value	β	*P*-value
LV dimensions																			
LV mass (g)	108.1 ± 18.2	−0.088	0.556	−0.111	0.464	−0.162	0.283	−0.205	0.167	−0.197	0.179	−0.163	0.275	−0.176	0.236	0.072	0.626	0.170	0.248
LV end-systolic volume (mL)	61.1 ± 14.3	−0.038	0.799	−0.103	0.496	−0.067	0.660	−0.114	0.444	−0.086	0.563	−0.025	0.867	−0.040	0.788	−0.220	0.132	−0.238	0.104
LV end-diastolic volume (mL)	155.7 ± 24.9	−0.149	0.316	−0.191	0.204	−0.179	0.234	-0.200	0.177	−0.156	0.288	−0.135	0.364	−0.170	0.253	−0.289	0.046*	−0.236	0.107
LVMV-ratio (g/mL)	0.7 ± 0.1	0.058	0.696	0.067	0.656	0.006	0.969	−0.011	0.940	−0.031	0.836	−0.035	0.813	−0.004	0.977	0.376	0.008*	0.375	0.009*
LV systolic function																			
LV Stroke volume (mL)	94.6 ± 17.1	−0.186	0.210	−0.189	0.209	−0.202	0.179	−0.196	0.188	−0.157	0.288	−0.176	0.235	−0.214	0.149	−0.238	0.104	−0.146	0.324
LV Ejection Fraction (%)	60.8 ± 5.8	−0.059	0.694	−0.001	0.994	−0.046	0.762	0.012	0.935	0.010	0.947	−0.068	0.650	−0.077	0.606	0.036	0.810	0.101	0.494
Cardiac index (L/min * m^−2^)	3022 ± 540	−0.058	0.697	−0.131	0.386	−0.117	0.440	−0.081	0.587	0.009	0.954	−0.057	0.705	−0.039	0.793	−0.165	0.262	−0.152	0.302
LV diastolic function																			
E peak filling rate (mL/s)	419.5 ± 82.6	−0.296	0.044*	−0.259	0.082	−0.262	0.078	−0.272	0.064	−0.185	0.209	−0.245	0.096	−0.256	0.082	−0.181	0.218	−0.234	0.110
E-dec_peak_ (mL/s^2^ *10^−3^)	−3.5 ± 1.0	0.335	0.022*	0.316	0.032*	0.334	0.023*	0.315	0.031*	0.266	0.067	0.279	0.058	0.300	0.040*	0.152	0.301	0.432	0.002*
E−dec_mean_ (mL/s^2^ *10^−3^)	−2.3 ± 0.6	0.459	0.001*	0.432	0.003*	0.435	0.003*	0.422	0.003*	0.338	0.019*	0.408	0.004*	0.422	0.003*	0.154	0.296	0.320	0.027*
E/A peak flow	1.0 ± 0.2	−0.413	0.004*	−0.366	0.012*	−0.381	0.009*	−0.338	0.020*	-0.333	0.021*	−0.355	0.014*	−0.380	0.008*	−0.118	0.423	−0.264	0.070
E/Ea	9.3 ± 3.4	0.022	0.891	0.092	0.563	0.083	0.608	0.070	0.660	−0.032	0.837	0.140	0.378	0.147	0.354	0.142	0.365	−0.108	0.489
Myocardial steatosis																			
Neutral lipid content (%)	0.9 ± 0.4	0.010	0.945	0.062	0.683	0.051	0.737	0.146	0.327	0.110	0.456	−0.014	0.927	−0.037	0.803	0.023	0.875	0.132	0.371

*Statistically significant. A: Atrial contraction; E: Early diastolic filling phase; E/Ea: Estimation of left ventricular filling pressures; LV: Left ventricular; LVMV: Left ventricular mass/left ventricular end-diastolic volume.

**Table 3:** Associations between clinical characteristics and circulating lncRNAs in patients with well-controlled type 2 diabetes.

	LIPCAR		uc004cos.4		uc004cov.4		uc004coz.1		uc011mfi.2		uc022bqu.1		uc022bqw.1		MIAT		SENCR	
	β	*P*-value	β	*P*-value	β	*P*-value	β	*P*-value	β	*P*-value	β	*P*-value	β	*P*-value	β	*P*-value	β	*P*-value
Age	0.372	0.010*	0.380	0.009*	0.304	0.040*	0.239	0.106	0.184	0.210	0.277	0.060	0.370	0.011*	0.165	0.261	0.162	0.271
Time since diagnosis of diabetes	0.373	0.010*	0.407	0.005*	0.375	0.010*	0.347	0.017*	0.289	0.047*	0.319	0.029*	0.301	0.040*	−0.071	0.633	0.190	0.195
BMI	−0.448	0.002*	−0.380	0.009*	−0.434	0.003*	−0.360	0.013*	−0.416	0.003*	−0.409	0.004*	−0.438	0.002*	−0.161	0.276	0.012	0.934
Waist circumference	−0.459	0.001*	−0.431	0.003*	−0.483	0.001*	−0.429	0.003*	−0.469	0.001*	−0.448	0.002*	-0.458	0.001*	−0.205	0.162	−0.001	0.995
Subcutaneous fat volume	−0.491	<0.001*	−0.416	0.004*	−0.453	0.002*	−0.404	0.005*	−0.462	0.001*	−0.453	0.001*	−0.468	0.001*	−0.168	0.255	−0.024	0.874
Plasma fasting insulin	−0.548	<0.001*	−0.468	0.001*	−0.472	0.001*	−0.441	0.002*	−0.527	<0.001*	−0.475	0.001*	−0.499	<0.001*	−0.165	0.268	0.067	0.654
HDL cholesterol	0.441	0.002*	0.337	0.023*	0.314	0.036*	0.299	0.043*	0.377	0.009*	0.342	0.020*	0.347	0.018*	0.122	0.413	−0.090	0.547
us−CRP	−0.307	0.038*	−0.271	0.071	−0.297	0.048*	−0.245	0.100	−0.317	0.030*	−0.334	0.023*	−0.308	0.037*	−0.148	0.321	−0.031	0.834

*Statistically significant.

**Table 4:** Association between LV diastolic function and circulating LIPCAR in patients with well-controlled type 2 diabetes.

	β	*P*-value
**Model 1**		
LIPCAR	−0.427	0.010*
Age	−0.192	0.191
BMI	−0.190	0.212
**Model 2**		
Association between E/A peak flow and circulating LIPCAR for model 1 and each of following variables:		
Plasma fasting glucose	−0.460	0.006*
Plasma fasting insulin	−0.436	0.018*
Time since diagnosis of diabetes	−0.423	0.015*
Myocardial Steatosis	−0.427	0.011*
HDL cholesterol	−0.372	0.034*
SBP	−0.442	0.008*
DBP	−0.386	0.021*
Heart rate	−0.480	0.004*
us-CRP	−0.399	0.024*
NT-proBNP	−0.448	0.009*

*Statistically significant.

**Table 5:** Association between LV diastolic dysfunction and circulating lncRNAs in patients with well-controlled type 2 diabetes.

	OR (95% IC)	*P*-value
**Univariate analysis**		
LIPCAR	1.047 (1.008, 1.087)	0.017*
uc004cos.4	1.033 (1.006, 1.062)	0.018*
uc004cov.4	1.172 (1.027, 1.337)	0.018*
uc004coz.1	1.040 (1.004, 1.077)	0.027*
uc011mfi.2	1.107 (1.019, 1.203)	0.017*
uc022bqw.1	1.043 (1.006, 1.082)	0.022*
uc022bqu.1	1.049 (1.008, 1.093)	0.020*
H19	1.959 (0.469, 9.178)	0.356
HOTAIR	1.067 (0.904, 1.259)	0.446
MIAT	0.890 (0.170, 4.666)	0.890
SENCR	1.673 (0.119, 3.545)	0.703
**Multivariate analysis**		
Model 1		
LIPCAR	1.053 (1.007, 1.101)	0.023*
Age	1.090 (0.957, 1.240)	0.195
BMI	1.171 (0.938, 1.462)	0.163
Model 2		
Association between LV diastolic dysfunction and circulating LIPCAR for model 1 and each of following variables:		
Plasma fasting glucose	1.058 (1.008, 1.111)	0.022*
Plasma fasting insulin	1.046 (1.002, 1.091)	0.040*
Time since diagnosis of diabetes	1.048 (1.003, 1.094)	0.034*
Myocardial Steatosis	1.054 (1.007, 1.103)	0.024*
HDL cholesterol	1.049 (1.005, 1.095)	0.030*
SBP	1.053 (1.007, 1.101)	0.022*
DBP	1.052 (1.006, 1.100)	0.025*
Heart rate	1.056 (1.008, 1.106)	0.021*
us-CRP	1.045 (1.001, 1.091)	0.044*
NT-proBNP	1.064 (1.013, 1.118)	0.013*

OR: Odds Ratio, CI: Confidence Interval. *Statistically significant.

**Table 6:** Association between LV remodelling and circulating lncRNAs in patients with well-controlled type 2 diabetes.

	β	*P*-value		β	*P*-value
**Model 1**			**Model 1**		
MIAT	0.396	0.005*	SENCR	0.345	0.015*
Age	0.172	0.211	Age	0.168	0.233
BMI	0.305	0.029*	BMI	0.236	0.092
**Model 2**			**Model 2**		
Association between LVMV-ratio and circulating MIAT for model 2 and each of following variables:			Association between LVMV-ratio and circulating SENCR for model 2 and each of following variables:		
Plasma fasting glucose	0.411	0.005*	Plasma fasting glucose	0.353	0.015*
Plasma fasting insulin	0.425	0.003*	Plasma fasting insulin	0.333	0.021*
Time since diagnosis of diabetes	0.404	0.005*	Time since diagnosis of diabetes	0.357	0.014*
SBP	0.385	0.007*	SBP	0.334	0.019*
DBP	0.375	0.008*	DBP	0.323	0.023*
Heart rate	0.390	0.006*	Heart rate	0.340	0.017*
Myocardial steatosis	0.396	0.006*	Myocardial steatosis	0.349	0.015*
NT-proBNP	0.401	0.005*	NT-proBNP	0.365	0.011*
us-CRP	0.389	0.008*	us-CRP	0.348	0.018*

*Statistically significant.

**Table 7:** Characteristics of the study population (Validation Study).

Variable	Type 2 diabetes Patients
	N = 30
Age (years)	55.0 ± 5.7
Time since diagnosis of diabetes (years)	4.3 ± 2.9
Body mass index (kg/m^2^)	28.0 ± 3.3
Waist circumference (cm)	101.1 ± 10.2
Subcutaneous fat volume (mL)	648.7 ± 272.6
Visceral fat volume (mL)	422.8 ± 207.8
Systolic blood pressure (mm Hg)	126.9 ± 11.0
Diastolic blood pressure (mmHg)	75.4 ± 8.6
Heart rate (bpm)	64.9 ± 9.4
Concomitant medication N (%)	
Statin	12 (40.0)
Any antihypertensive medication	13 (43.3)
β-Blocker	1 (3.3)
Diuretic	1 (3.3)
ACE inhibitor	8 (26.7)
ARB	4 (13.3)
Calcium antagonist	1 (3.3)
HbA_1c_ (%)	7.0 ± 1.0
Plasma fasting glucose (mmol/L)	7.8 ± 1.3
Plasma fasting insulin (pmol/L)	79.7 ± 45.6
Total cholesterol (mmol/L)	4.9 ± 1.2
LDL cholesterol (mmol/L)	2.8 ± 0.8
HDL cholesterol (mmol/L)	1.1 ± 0.3
Plasma triglycerides (mmol/L)	2.3 ± 2.8
Plasma NEFA (mmol/L)	0.5 ± 0.2
us-CRP (mg/L)	12.8 ± 29.3
NT-proBNP (ng/L)	30.9 ± 21.7
Parameters of cardiac dimensions and function	
LV mass (g)	106.1 ± 14.3
LV end-systolic volume (mL)	65.5 ± 14.8
LV end-diastolic volume (mL)	157.6 ± 25.1
LVMV-ratio (g/mL)	0.7 ± 0.1
LV Stroke volume (mL)	92.2 ± 14.5
LV Ejection Fraction (%)	58.6 ± 5.1
Cardiac index (L/min * m^−2^)	2789.9 ± 438.8
E peak filling rate (mL/s)	412.1 ± 82.3
E-dec_peak_ (mL/s^2^*10^−3^)	−3.4 ± 1.0
E-dec_mean_ (mL/s^2^*10^−3^)	−2.2 ± 0.7
E/A peak flow	1.1 ± 0.3
E/Ea	10.6 ± 4.6
Neutral lipid content (%)	0.8 ± 0.5

Data are presented as mean ± SD for continuous variables and as frequencies (percentages) for categorical variables. *Statistically significant. ACE: Angiotensin-converting enzyme; ARB: angiotensin receptor blocker; HbA_1c_: Glycated haemoglobin. For other abbreviations see the text.

**Table 8:** Association between LV diastolic function/remodelling and circulating lncRNAs in patients with well-controlled type 2 diabetes (Validation Study).

	β	*P*-value
E/A peak flow		
LIPCAR	−0.593	0.001*
LVMV-ratio		
MIAT	0.471	0.009*
SENCR	0.421	0.021*

*Statistically significant.
