# Supplementary material for: Circulating long-non coding RNAs as biomarkers of left ventricular diastolic function and remodelling in patients with well-controlled type 2 diabetes
Source: Sci Rep. 2016 Nov 22;6:37354. doi: 10.1038/srep37354 (PMC5118808; doi:10.1038/srep37354)
Supplement: Supplementary material [file srep37354-s1.pdf]

Circulating long-non coding RNAs as biomarkers of left ventricular diastolic function and remodelling in patients with well-controlled type 2 diabetes

de Gonzalo-Calvo D,[1 2 *] Kenneweg F,[2] Bang C,[2] Toro R,[3] van der Meer RW,[4] Rijzewijk LJ,[5] Smit JW,[6] Lamb HJ,[4] Llorente-Cortes V,[1 * #] Thum T [2 7 8 #]

[1] Cardiovascular Research Center (CSIC-ICCC), Biomedical Research Institute Sant Pau (IIB Sant Pau), Barcelona, Spain.

[2] Institute of Molecular and Translational Therapeutic Strategies (IMTTS), IFB-Tx, Hannover Medical School, Hannover, Germany.

[3] Department of Medicine, University of Cádiz, Spain.

[4] Department of Radiology, Leiden University Medical Center, Leiden, the Netherlands.

[5] Department of Medicine, Kantonsspital Baden AG, Baden, Switzerland.

[6] Department of Internal Medicine, University Medical Center Nijmegen, Nijmegen, the Netherlands.

[7] Excellence Cluster REBIRTH, Hannover Medical School, Hannover, Germany.

[8] National Heart and Lung Institute, Imperial College London, UK.

# These authors contributed equally to this article.

*** Corresponding authors:**

David de Gonzalo Calvo, PhD

Cardiovascular Research Center (CSIC-ICCC), Biomedical Research Institute Sant Pau (IIB Sant Pau), Av. Sant Antoni Maria Claret 167, Pavelló del Convent, 08025 Barcelona, Spain.

Tel: +34 93 556 5901, Fax: +34 935565559

E-mail: david.degonzalo@gmail.com / DGonzalo@santpau.cat

Vicenta Llorente Cortés, PhD

Cardiovascular Research Center (CSIC-ICCC), Biomedical Research Institute Sant Pau (IIB Sant Pau), Av. Sant Antoni Maria Claret 167, Pavelló del Convent, 08025 Barcelona, Spain.

Tel: +34 93 556 5888, Fax: +34 935565559

E-mail: cllorente@csic-iccc.org

SUPPLEMENTARY TABLES

Table S1. Correlations between circulating lncRNAs in patients with well-controlled type 2 diabetes.

		uc004cos.4	uc004cov.4	uc004coz.1	uc011mfi.2	uc022bqu.1	uc022bqw.1	H19	HOTAIR	MIAT	SENCR
**LIPCAR**	ρ	0.964	0.966	0.915	0.850	0.971	0.964	-0.034	0.037	0.320	0.173
	P-value	<0.001*	<0.001*	<0.001*	<0.001*	<0.001*	<0.001*	0.828	0.805	0.028*	0.246
**uc004cos.4**	ρ		0.978	0.924	0.791	0.966	0.964	0.019	-0.096	0.358	0.289
	P-value		<0.001*	<0.001*	<0.001*	<0.001*	<0.001*	0.905	0.526	0.015*	0.051
**uc004cov.4**	ρ			0.949	0.843	0.977	0.963	0.003	-0.060	0.352	0.327
	P-value			<0.001*	<0.001*	<0.001*	<0.001*	0.983	0.690	0.017*	0.027*
**uc004coz.1**	ρ				0.844	0.937	0.897	0.027	-0.098	0.385	0.167
	P-value				<0.001*	<0.001*	<0.001*	0.861	0.511	0.008*	0.262
**uc011mfi.2**	ρ					0.830	0.797	0.003	0.038	0.260	0.128
	P-value					<0.001*	<0.001*	0.985	0.800	0.074	0.387
**uc022bqu.1**	ρ						0.970	-0.059	-0.108	0.325	0.144
	P-value						<0.001*	0.705	0.468	0.026*	0.333
**uc022bqw.1**	ρ							-0.070	-0.105	0.334	0.159
	P-value							0.654	0.482	0.022*	0.285
**H19**	ρ								0.517	0.443	0.476
	P-value								<0.001*	0.002*	<0.001*
**HOTAIR**	ρ									0.418	0.416
	P-value									0.003*	0.003*
**MIAT**	ρ										0.347
	P-value										0.016*

*: Statistically significant.

Table S2. Correlations between circulating lncRNAs in healthy volunteers.

		uc004cos.4	uc004cov.4	uc004coz.1	uc011mfi.2	uc022bqu.1	uc022bqw.1	H19	MIAT	HOTAIR	SENCR
**LIPCAR**	ρ	0.930	0.963	0.870	0.936	0.960	0.954	0.024	0.324	0.187	-0.226
	P-value	<0.001*	<0.001*	<0.001*	<0.001*	<0.001*	<0.001*	0.947	0.330	0.583	0.531
**uc004cos.4**	ρ		0.979	0.893	0.965	0.921	0.978	0.121	0.122	0.222	-0.055
	P-value		<0.001*	<0.001*	<0.001*	<0.001*	<0.001*	0.739	0.720	0.511	0.880
**uc004cov.4**	ρ			0.937	0.984	0.966	0.993	0.148	0.281	0.141	0.000
	P-value			<0.001*	<0.001*	<0.001*	<0.001*	0.683	0.432	0.697	1.000
**uc004coz.1**	ρ				0.930	0.920	0.937	0.316	0.326	0.219	-0.058
	P-value				<0.001*	<0.001*	<0.001*	0.373	0.328	0.517	0.873
**uc011mfi.2**	ρ					0.975	0.968	0.139	0.207	0.182	-0.085
	P-value					<0.001*	<0.001*	0.703	0.541	0.592	0.816
**uc022bqu.1**	ρ						0.942	0.113	0.402	0.119	-0.058
	P-value						<0.001*	0.755	0.249	0.744	0.882
**uc022bqw.1**	ρ							0.210	0.251	0.196	-0.087
	P-value							0.560	0.456	0.563	0.811
**H19**	ρ								0.544	0.655	0.452
	P-value								0.083	0.029*	0.189
**HOTAIR**	ρ									0.206	0.150
	P-value									0.521	0.659
**MIAT**	ρ										0.330
	P-value										0.321

*: Statistically significant.

Table S3. Area under the ROC curve for models of grade I diastolic dysfunction in patients with well-controlled type 2 diabetes.

	AUC (95% IC)
**Univariate analysis**	
LIPCAR	0.745 (0.599, 0.891)
**Multivariate analysis**	
**Model 1**	0.794 (0.659, 0.930)
LIPCAR	
Age	
BMI	
**Model 2**	
AUC for model 1 and each of following variables:	
Plasma fasting glucose	0.842 (0.725, 0.959)
Plasma fasting insulin	0.822 (0.697, 0.948)
Time since diagnosis of diabetes	0.804 (0.673, 0.936)
Myocardial Steatosis	0.808 (0.677, 0.939)
HDL cholesterol	0.814 (0.683, 0.946)
SBP	0.791 (0.653, 0.928)
DBP	0.798 (0.661, 0.936)
Heart rate	0.810 (0.674, 0.946)
us-CRP	0.818 (0.693, 0.944)
NT-proBNP	0.820 (0.690, 0.950)

AUC: Area under the ROC curve, CI: Confidence Interval.

*: Statistically significant.

Table S4. Characteristics of the initial and validation groups.

Variable	Initial study N = 48	Validation Study N = 30	*P*-value
Age (years)	57.5 ± 5.4	55.0 ± 5.7	0.048*
Time since diagnosis of diabetes (years)	4.0 ± 2.5	4.3 ± 2.9	0.569
Body mass index (kg/m^2)	29.2 ± 3.5	28.0 ± 3.3	0.140
Waist circumference (cm)	106.4 ± 9.8	101.1 ± 10.2	0.024*
Subcutaneous fat volume (mL)	706.6 ± 248.4	648.7 ± 272.6	0.338
Visceral fat volume (mL)	444.65 ± 206.6	422.8 ± 207.8	0.652
Systolic blood pressure (mm Hg)	128.1 ± 12.2	126.9 ± 11.0	0.683
Diastolic blood pressure (mmHg)	75.9 ± 6.1	75.4 ± 8.6	0.729
Heart rate (bpm)	65.7 ± 8.5	64.9 ± 9.4	0.709
Concomitant medication N (%)			
Statin	25 (52.1)	12 (40.0)	0.210
Any antihypertensive medication	21 (43.8)	13 (43.3)	0.579
β-Blocker	6 (12.5)	1 (3.3)	0.167
Diuretic	11 (22.9)	1 (3.3)	0.017*
ACE inhibitor	10 (20.8)	8 (26.7)	0.371
ARB	5 (10.4)	4 (13.3)	0.480
Calcium antagonist	3 (6.3)	1 (3.3)	0.500
HbA_{1c} (%)	7.2 ± 1.0	7.0 ± 1.0	0.321
Plasma fasting glucose (mmol/L)	9.0 ± 2.1	7.8 ± 1.3	0.003*
Plasma fasting insulin (pmol/L)	66.8 ± 35.4	79.7 ± 45.6	0.168
Total cholesterol (mmol/L)	4.6 ± 0.9	4.9 ± 1.2	0.242
LDL cholesterol (mmol/L)	2.7 ± 0.8	2.8 ± 0.8	0.574
HDL cholesterol (mmol/L)	1.1 ± 0.3	1.1 ± 0.3	0.983
Plasma triglycerides (mmol/L)	1.8 ± 1.1	2.3 ± 2.8	0.247
Plasma NEFA (mmol/L)	0.5 ± 0.2	0.5 ± 0.2	0.634
us-CRP (mg/L)	5.3 ± 4.2	12.8 ± 29.3	0.175
NT-proBNP (ng/L)	38.4 ± 29.8	30.9 ± 21.7	0.265
Parameters of cardiac dimensions and function			
LV mass (g)	108.1 ± 18.2	106.1 ± 14.3	0.604
LV end-systolic volume (mL)	61.1 ± 14.3	65.5 ± 14.8	0.195
LV end-diastolic volume (mL)	155.7 ± 24.9	157.6 ± 25.1	0.740
LVMV-ratio (g/mL)	0.7 ± 0.1	0.7 ± 0.1	0.516
LV Stroke volume (mL)	94.6 ± 17.1	92.2 ± 14.5	0.514
LV Ejection Fraction (%)	60.8 ± 5.8	58.6 ± 5.1	0.096
Cardiac index ($L/min * m^{-2}$)	3022 ± 540	2789.9 ± 438.8	0.058
E peak filling rate (mL/s)	419.5 ± 82.6	412.1 ± 82.3	0.707
$E\text{-dec}_{peak}$ ($mL/s^2 *10^{-3}$)	-3.5 ± 1.0	-3.4 ± 1.0	0.529
$E\text{-dec}_{mean}$ ($mL/s^2 *10^{-3}$)	-2.3 ± 0.6	-2.2 ± 0.7	0.369
E/A peak flow	1.0 ± 0.2	1.1 ± 0.3	0.879

E/Ea	9.3 ± 3.4	10.6 ± 4.6	0.176
Neutral lipid content (%)	0.9 ± 0.4	0.8 ± 0.5	0.587

Data are presented as mean ± SD for continuous variables and as frequencies (percentages) for categorical variables.
*: Statistically significant.
ACE: Angiotensin-converting enzyme; ARB: angiotensin receptor blocker; HbA_{1c}: Glycated haemoglobin. For other abbreviations see the text.

Table S5. Oligonucleotide sequences used for lncRNA detection.

LncRNA	Forward primer	Reverse primer	Hybridization Temperature (ºC)	Fragment size
LIPCAR (uc022bqs.1)	TAAAGGATGCGTAGGGATGG	TTCATGATCACGCCCTCATA	58ºC	187
uc004cos.4	ATGGCCAACCTCCTACTCCT	TAGATGTGGCGGGTTTTAGG	58ºC	193
uc004cov.4	TTCCCCAACCTTTTCCTCCG	TGGATAAGTGGCGTTGGCTT	58ºC	101
uc004coz.1	CAAATCCCTTCTCGTCCCCA	TACCCCCAAGTGTTATGGGC	58ºC	129
uc011mfi.2	ACCGGGGGTATACTACGGTC	GCTCTAGAGGGGGTAGAGGG	58ºC	145
uc022bqw.1	TATCCGCCATCCCATACATT	GGTGATTCCTAGGGGGTTGT	58ºC	188
uc022bqu.1	GCGGCTTCGACCCTATATCC	AGGGCTCATGGTAGGGGTAA	58ºC	121
H19	AGACAGTACAGCATCCAGGG	GAGACCTGGCCTCGTCTC	60ºC	239
HOTAIR	GCCAGTACCGACCTGGTAGA	TGTCTGTGAGTGCCCGTCT	60ºC	173
MALAT1	TGTGTGCCAATGTTTCGTTT	AGGAGAAAGTGCCATGGTTG	58ºC	200
MIAT	TGTCTCCATTTGCTCAGTGC	TCAGGATGGTGCACTCTCAG	58ºC	190
SENCR	CAGCCAGAAAGGACTCCAACTCC	GGAGGCAGCTGGTGCTGAAAG	60ºC	273

SUPPLEMENTARY FIGURES

FIGURE S1

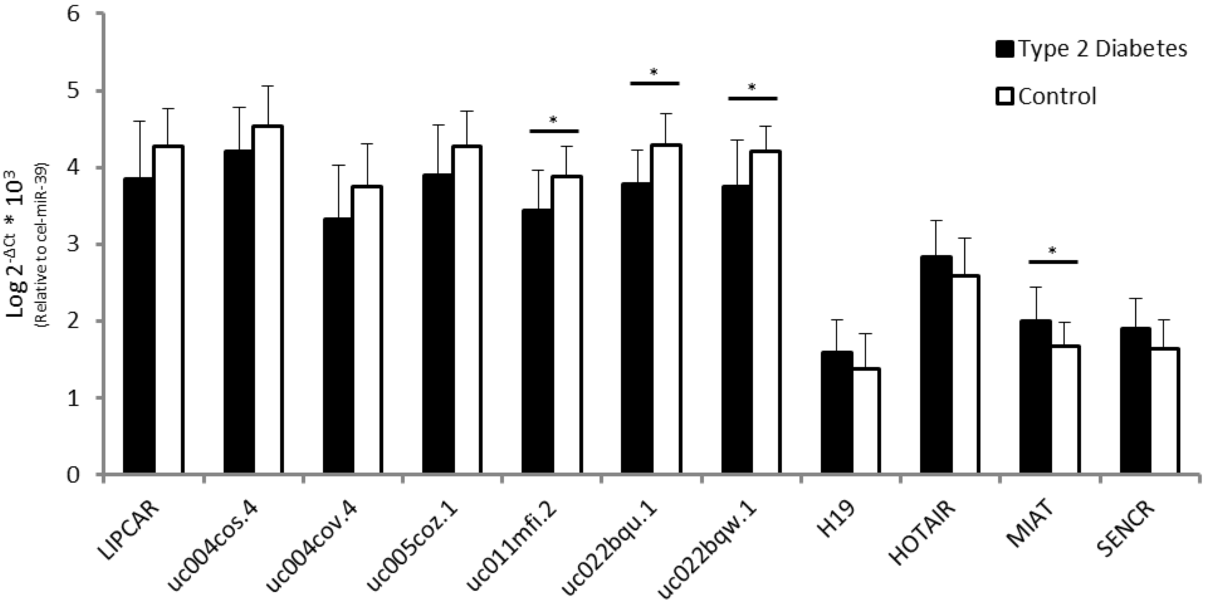

Circulating lncRNA expression profile in patients with well-controlled type 2 diabetes and healthy age-matched volunteers. Bar graphs show the mean ± SD. Results are expressed as arbitrary units. * $P < 0.050$.

FIGURE S2

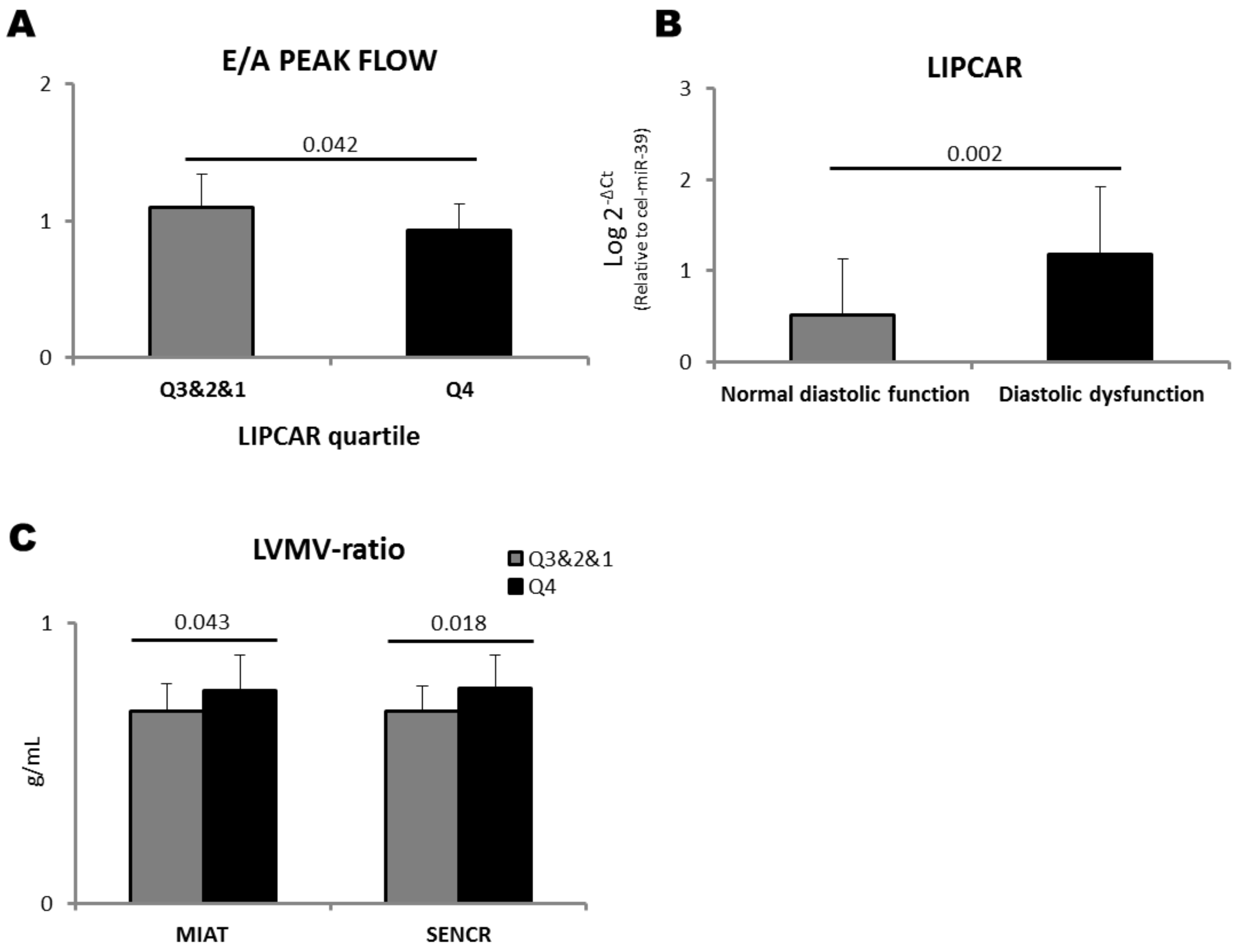

A) E/A peak flow ratio in type 2 diabetes patients with serum LIPCAR levels in quartiles 1, 2 and 3, or 4; B) Real time quantification of serum LIPCAR in type 2 diabetes patients with normal diastolic function and grade I diastolic dysfunction; C) LVMV-ratio in type 2 diabetes patients with serum MIAT and SENCR levels in quartiles 1, 2 and 3, or 4. Bar graphs show the mean ± SD.

FIGURE S3

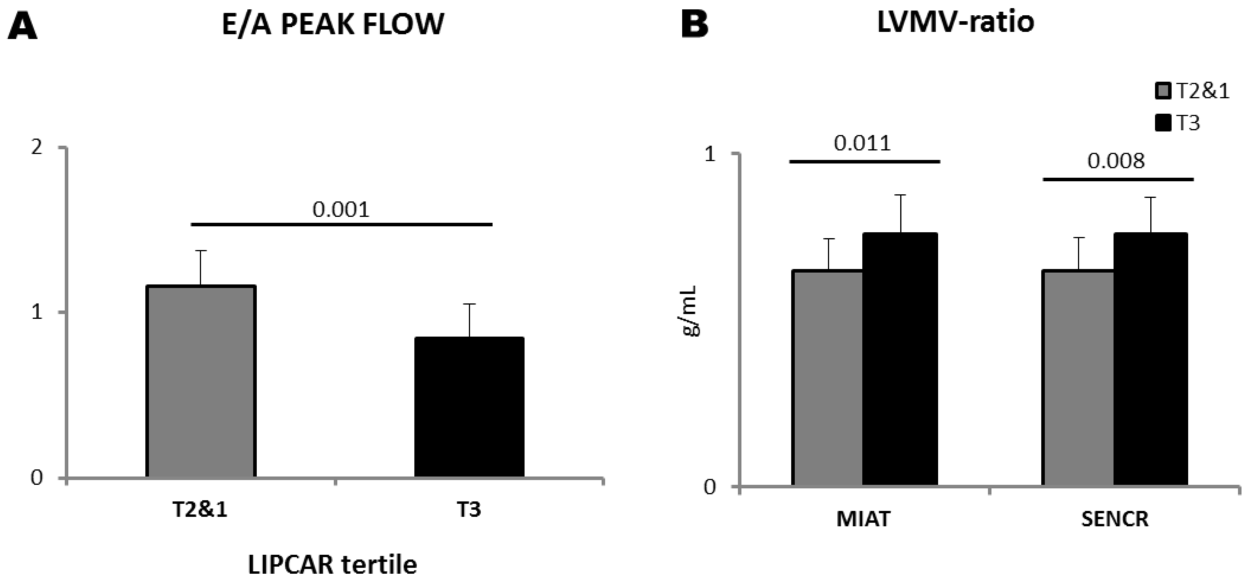

A) E/A peak flow ratio in type 2 diabetes patients with serum LIPCAR levels in tertiles 1 and 2 or 3 (Validation study); B) LVMV-ratio in type 2 diabetes patients with serum MIAT and SENCR levels in tertiles 1 and 2 or 3 (Validation study). Bar graphs show the mean ± SD.

FIGURE S4

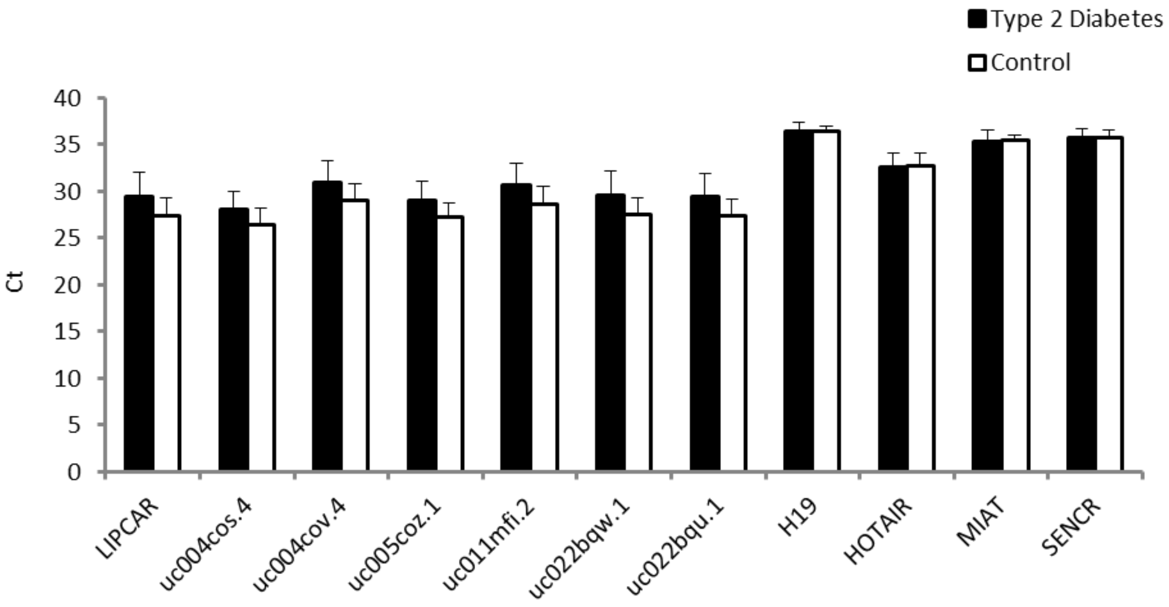

Expression levels of circulating lncRNAs in serum of patients with well-controlled type 2 diabetes and healthy age-matched volunteers. Bar graphs show the mean ± SD.
